# Supplementary material for: Transcriptome Analysis Reveals Vimentin-Induced Disruption of Cell–Cell Associations Augments Breast Cancer Cell Migration
Source: Cells. 2022 Dec 13;11(24):4035. doi: 10.3390/cells11244035 (PMC9776984; doi:10.3390/cells11244035)
Supplement: Supplementary file 1 [file cells-11-04035-s001.zip › DEGs and enrichment analysis.zip!/DEGs and enrichment analysis/WT_VIMvsVector KEGG/DOWN/WT_VIMvsVector_DOWN.pathway_map.zip!/WT_VIMvsVector_DOWN.pathway_map/pathway/WT_VIMvsVector.html]

WT\_VIMvsVector.utf8.md


###WT\_VIMvsVector KEGG通路富集列表 \*\*\*

| KEGGID | Description | GeneRatio | BgRatio | pvalue | padj | geneID | geneName | keggID | Count |
| --- | --- | --- | --- | --- | --- | --- | --- | --- | --- |
| hsa05146 | Amoebiasis | 9\|118 | 94\|9228 | 0.0000029 | 0.0005899 | ENSG00000169429/ENSG00000058085/ENSG00000092969/ENSG00000188153/ENSG00000169031/ENSG00000081041/ENSG00000135862/ENSG00000053747/ENSG00000137462 | CXCL8/LAMC2/TGFB2/COL4A5/COL4A3/CXCL2/LAMC1/LAMA3/TLR2 | hsa:3576/hsa:3918/hsa:7042/hsa:1287/hsa:1285/hsa:2920/hsa:3915/hsa:3909/hsa:7097 | 9 |
| hsa00830 | Retinol metabolism | 5\|118 | 57\|9228 | 0.0007736 | 0.0672460 | ENSG00000162496/ENSG00000128918/ENSG00000003137/ENSG00000256612/ENSG00000095596 | DHRS3/ALDH1A2/CYP26B1/CYP2B7P/CYP26A1 | hsa:9249/hsa:8854/hsa:56603/hsa:1555/hsa:1592 | 5 |
| hsa04512 | ECM-receptor interaction | 6\|118 | 91\|9228 | 0.0010595 | 0.0672460 | ENSG00000058085/ENSG00000188153/ENSG00000169031/ENSG00000135862/ENSG00000053747/ENSG00000115221 | LAMC2/COL4A5/COL4A3/LAMC1/LAMA3/ITGB6 | hsa:3918/hsa:1287/hsa:1285/hsa:3915/hsa:3909/hsa:3694 | 6 |
| hsa00982 | Drug metabolism - cytochrome P450 | 5\|118 | 64\|9228 | 0.0013121 | 0.0672460 | ENSG00000213366/ENSG00000259583/ENSG00000184254/ENSG00000256612/ENSG00000241720 | GSTM2/AC015712.2/ALDH1A3/CYP2B7P/AL158847.1 | hsa:2946/hsa:220/hsa:220/hsa:1555/hsa:2947 | 5 |
| hsa00980 | Metabolism of xenobiotics by cytochrome P450 | 5\|118 | 69\|9228 | 0.0018386 | 0.0753834 | ENSG00000213366/ENSG00000259583/ENSG00000184254/ENSG00000256612/ENSG00000241720 | GSTM2/AC015712.2/ALDH1A3/CYP2B7P/AL158847.1 | hsa:2946/hsa:220/hsa:220/hsa:1555/hsa:2947 | 5 |
| hsa04621 | NOD-like receptor signaling pathway | 8\|118 | 195\|9228 | 0.0034710 | 0.1166698 | ENSG00000169429/ENSG00000117226/ENSG00000162645/ENSG00000139112/ENSG00000111335/ENSG00000081041/ENSG00000023445/ENSG00000132357 | CXCL8/GBP3/GBP2/GABARAPL1/OAS2/CXCL2/BIRC3/CARD6 | hsa:3576/hsa:2635/hsa:2634/hsa:23710/hsa:4939/hsa:2920/hsa:330/hsa:84674 | 8 |
| hsa05222 | Small cell lung cancer | 6\|118 | 121\|9228 | 0.0044843 | 0.1166698 | ENSG00000058085/ENSG00000188153/ENSG00000169031/ENSG00000135862/ENSG00000053747/ENSG00000023445 | LAMC2/COL4A5/COL4A3/LAMC1/LAMA3/BIRC3 | hsa:3918/hsa:1287/hsa:1285/hsa:3915/hsa:3909/hsa:330 | 6 |
| hsa04510 | Focal adhesion | 9\|118 | 249\|9228 | 0.0045530 | 0.1166698 | ENSG00000058085/ENSG00000128052/ENSG00000162909/ENSG00000188153/ENSG00000169031/ENSG00000135862/ENSG00000053747/ENSG00000023445/ENSG00000115221 | LAMC2/KDR/CAPN2/COL4A5/COL4A3/LAMC1/LAMA3/BIRC3/ITGB6 | hsa:3918/hsa:3791/hsa:824/hsa:1287/hsa:1285/hsa:3915/hsa:3909/hsa:330/hsa:3694 | 9 |
| hsa05145 | Toxoplasmosis | 6\|118 | 125\|9228 | 0.0052526 | 0.1196417 | ENSG00000058085/ENSG00000092969/ENSG00000135862/ENSG00000053747/ENSG00000023445/ENSG00000137462 | LAMC2/TGFB2/LAMC1/LAMA3/BIRC3/TLR2 | hsa:3918/hsa:7042/hsa:3915/hsa:3909/hsa:330/hsa:7097 | 6 |
| hsa05144 | Malaria | 3\|118 | 33\|9228 | 0.0084106 | 0.1724166 | ENSG00000169429/ENSG00000092969/ENSG00000137462 | CXCL8/TGFB2/TLR2 | hsa:3576/hsa:7042/hsa:7097 | 3 |
| hsa04933 | AGE-RAGE signaling pathway in diabetic complications | 5\|118 | 102\|9228 | 0.0097738 | 0.1821478 | ENSG00000078401/ENSG00000169429/ENSG00000092969/ENSG00000188153/ENSG00000169031 | EDN1/CXCL8/TGFB2/COL4A5/COL4A3 | hsa:1906/hsa:3576/hsa:7042/hsa:1287/hsa:1285 | 5 |
| hsa05204 | Chemical carcinogenesis | 4\|118 | 72\|9228 | 0.0134096 | 0.2290812 | ENSG00000213366/ENSG00000259583/ENSG00000184254/ENSG00000241720 | GSTM2/AC015712.2/ALDH1A3/AL158847.1 | hsa:2946/hsa:220/hsa:220/hsa:2947 | 4 |
| hsa05323 | Rheumatoid arthritis | 4\|118 | 75\|9228 | 0.0153889 | 0.2426711 | ENSG00000169429/ENSG00000092969/ENSG00000081041/ENSG00000137462 | CXCL8/TGFB2/CXCL2/TLR2 | hsa:3576/hsa:7042/hsa:2920/hsa:7097 | 4 |
| hsa05321 | Inflammatory bowel disease (IBD) | 3\|118 | 44\|9228 | 0.0184499 | 0.2701591 | ENSG00000092969/ENSG00000138378/ENSG00000137462 | TGFB2/STAT4/TLR2 | hsa:7042/hsa:6775/hsa:7097 | 3 |
| hsa04151 | PI3K-Akt signaling pathway | 10\|118 | 376\|9228 | 0.0219792 | 0.2739831 | ENSG00000157613/ENSG00000058085/ENSG00000128052/ENSG00000109321/ENSG00000188153/ENSG00000169031/ENSG00000135862/ENSG00000053747/ENSG00000115221/ENSG00000137462 | CREB3L1/LAMC2/KDR/AREG/COL4A5/COL4A3/LAMC1/LAMA3/ITGB6/TLR2 | hsa:90993/hsa:3918/hsa:3791/hsa:374/hsa:1287/hsa:1285/hsa:3915/hsa:3909/hsa:3694/hsa:7097 | 10 |
| hsa04080 | Neuroactive ligand-receptor interaction | 7\|118 | 219\|9228 | 0.0220789 | 0.2739831 | ENSG00000078401/ENSG00000135346/ENSG00000136928/ENSG00000148926/ENSG00000168398/ENSG00000065325/ENSG00000164418 | EDN1/CGA/GABBR2/ADM/BDKRB2/GLP2R/GRIK2 | hsa:1906/hsa:1081/hsa:9568/hsa:133/hsa:624/hsa:9340/hsa:2898 | 7 |
| hsa04514 | Cell adhesion molecules (CAMs) | 5\|118 | 128\|9228 | 0.0239465 | 0.2739831 | ENSG00000179776/ENSG00000173482/ENSG00000134258/ENSG00000107738/ENSG00000188404 | CDH5/PTPRM/VTCN1/VSIR/SELL | hsa:1003/hsa:5797/hsa:79679/hsa:64115/hsa:6402 | 5 |
| hsa00360 | Phenylalanine metabolism | 2\|118 | 19\|9228 | 0.0240570 | 0.2739831 | ENSG00000259583/ENSG00000184254 | AC015712.2/ALDH1A3 | hsa:220/hsa:220 | 2 |
| hsa04926 | Relaxin signaling pathway | 5\|118 | 133\|9228 | 0.0276806 | 0.2986591 | ENSG00000078401/ENSG00000157613/ENSG00000188153/ENSG00000169031/ENSG00000137745 | EDN1/CREB3L1/COL4A5/COL4A3/MMP13 | hsa:1906/hsa:90993/hsa:1287/hsa:1285/hsa:4322 | 5 |
| hsa05142 | Chagas disease (American trypanosomiasis) | 4\|118 | 93\|9228 | 0.0310765 | 0.3185342 | ENSG00000169429/ENSG00000092969/ENSG00000168398/ENSG00000137462 | CXCL8/TGFB2/BDKRB2/TLR2 | hsa:3576/hsa:7042/hsa:624/hsa:7097 | 4 |
| hsa05418 | Fluid shear stress and atherosclerosis | 6\|118 | 188\|9228 | 0.0333218 | 0.3252839 | ENSG00000179776/ENSG00000078401/ENSG00000213366/ENSG00000128052/ENSG00000116701/ENSG00000241720 | CDH5/EDN1/GSTM2/KDR/NCF2/AL158847.1 | hsa:1003/hsa:1906/hsa:2946/hsa:3791/hsa:4688/hsa:2947 | 6 |
| hsa04270 | Vascular smooth muscle contraction | 5\|118 | 146\|9228 | 0.0390577 | 0.3580035 | ENSG00000078401/ENSG00000185532/ENSG00000122786/ENSG00000148926/ENSG00000188257 | EDN1/PRKG1/CALD1/ADM/PLA2G2A | hsa:1906/hsa:5592/hsa:800/hsa:133/hsa:5320 | 5 |
| hsa00340 | Histidine metabolism | 2\|118 | 25\|9228 | 0.0401662 | 0.3580035 | ENSG00000259583/ENSG00000184254 | AC015712.2/ALDH1A3 | hsa:220/hsa:220 | 2 |
| hsa04061 | Viral protein interaction with cytokine and cytokine receptor | 3\|118 | 61\|9228 | 0.0429327 | 0.3667165 | ENSG00000169429/ENSG00000006606/ENSG00000081041 | CXCL8/CCL26/CXCL2 | hsa:3576/hsa:10344/hsa:2920 | 3 |
| hsa05202 | Transcriptional misregulation in cancer | 6\|118 | 205\|9228 | 0.0474949 | 0.3894584 | ENSG00000164120/ENSG00000169429/ENSG00000146674/ENSG00000023445/ENSG00000058091/ENSG00000132170 | HPGD/CXCL8/IGFBP3/BIRC3/CDK14/PPARG | hsa:3248/hsa:3576/hsa:3486/hsa:330/hsa:5218/hsa:5468 | 6 |
| hsa04024 | cAMP signaling pathway | 6\|118 | 214\|9228 | 0.0563138 | 0.4414000 | ENSG00000125257/ENSG00000078401/ENSG00000157613/ENSG00000135346/ENSG00000136928/ENSG00000125398 | ABCC4/EDN1/CREB3L1/CGA/GABBR2/SOX9 | hsa:10257/hsa:1906/hsa:90993/hsa:1081/hsa:9568/hsa:6662 | 6 |
| hsa04216 | Ferroptosis | 3\|118 | 69\|9228 | 0.0581356 | 0.4414000 | ENSG00000047457/ENSG00000130066/ENSG00000240216 | CP/SAT1/CPHL1P | hsa:1356/hsa:6303/hsa:1356 | 3 |
| hsa04610 | Complement and coagulation cascades | 3\|118 | 71\|9228 | 0.0622845 | 0.4560114 | ENSG00000237248/ENSG00000175899/ENSG00000168398 | LINC00987/A2M/BDKRB2 | hsa:2/hsa:2/hsa:624 | 3 |
| hsa00410 | beta-Alanine metabolism | 2\|118 | 34\|9228 | 0.0697746 | 0.4932339 | ENSG00000259583/ENSG00000184254 | AC015712.2/ALDH1A3 | hsa:220/hsa:220 | 2 |
| hsa04668 | TNF signaling pathway | 4\|118 | 126\|9228 | 0.0777791 | 0.5137570 | ENSG00000078401/ENSG00000157613/ENSG00000081041/ENSG00000023445 | EDN1/CREB3L1/CXCL2/BIRC3 | hsa:1906/hsa:90993/hsa:2920/hsa:330 | 4 |
| hsa05161 | Hepatitis B | 5\|118 | 179\|9228 | 0.0792298 | 0.5137570 | ENSG00000157613/ENSG00000169429/ENSG00000092969/ENSG00000138378/ENSG00000137462 | CREB3L1/CXCL8/TGFB2/STAT4/TLR2 | hsa:90993/hsa:3576/hsa:7042/hsa:6775/hsa:7097 | 5 |
| hsa05140 | Leishmaniasis | 3\|118 | 79\|9228 | 0.0801962 | 0.5137570 | ENSG00000092969/ENSG00000116701/ENSG00000137462 | TGFB2/NCF2/TLR2 | hsa:7042/hsa:4688/hsa:7097 | 3 |
| hsa00350 | Tyrosine metabolism | 2\|118 | 39\|9228 | 0.0884922 | 0.5497240 | ENSG00000259583/ENSG00000184254 | AC015712.2/ALDH1A3 | hsa:220/hsa:220 | 2 |
| hsa01524 | Platinum drug resistance | 3\|118 | 88\|9228 | 0.1026863 | 0.6115209 | ENSG00000213366/ENSG00000023445/ENSG00000241720 | GSTM2/BIRC3/AL158847.1 | hsa:2946/hsa:330/hsa:2947 | 3 |
| hsa00860 | Porphyrin and chlorophyll metabolism | 2\|118 | 43\|9228 | 0.1044060 | 0.6115209 | ENSG00000047457/ENSG00000240216 | CP/CPHL1P | hsa:1356/hsa:1356 | 2 |
| hsa04012 | ErbB signaling pathway | 3\|118 | 94\|9228 | 0.1189184 | 0.6361595 | ENSG00000109321/ENSG00000174808/ENSG00000114423 | AREG/BTC/CBLB | hsa:374/hsa:685/hsa:868 | 3 |
| hsa04657 | IL-17 signaling pathway | 3\|118 | 94\|9228 | 0.1189184 | 0.6361595 | ENSG00000169429/ENSG00000137745/ENSG00000081041 | CXCL8/MMP13/CXCL2 | hsa:3576/hsa:4322/hsa:2920 | 3 |
| hsa04218 | Cellular senescence | 5\|118 | 204\|9228 | 0.1202767 | 0.6361595 | ENSG00000169429/ENSG00000146674/ENSG00000162909/ENSG00000092969/ENSG00000185650 | CXCL8/IGFBP3/CAPN2/TGFB2/ZFP36L1 | hsa:3576/hsa:3486/hsa:824/hsa:7042/hsa:677 | 5 |
| hsa04962 | Vasopressin-regulated water reabsorption | 2\|118 | 47\|9228 | 0.1210255 | 0.6361595 | ENSG00000157613/ENSG00000111348 | CREB3L1/ARHGDIB | hsa:90993/hsa:397 | 2 |
| hsa04060 | Cytokine-cytokine receptor interaction | 5\|118 | 208\|9228 | 0.1276324 | 0.6511849 | ENSG00000181634/ENSG00000169429/ENSG00000092969/ENSG00000006606/ENSG00000081041 | TNFSF15/CXCL8/TGFB2/CCL26/CXCL2 | hsa:9966/hsa:3576/hsa:7042/hsa:10344/hsa:2920 | 5 |
| hsa04064 | NF-kappa B signaling pathway | 3\|118 | 98\|9228 | 0.1302370 | 0.6511849 | ENSG00000169429/ENSG00000081041/ENSG00000023445 | CXCL8/CXCL2/BIRC3 | hsa:3576/hsa:2920/hsa:330 | 3 |
| hsa02010 | ABC transporters | 2\|118 | 51\|9228 | 0.1382411 | 0.6737286 | ENSG00000125257/ENSG00000198691 | ABCC4/ABCA4 | hsa:10257/hsa:24 | 2 |
| hsa00072 | Synthesis and degradation of ketone bodies | 1\|118 | 12\|9228 | 0.1431807 | 0.6737286 | ENSG00000134240 | HMGCS2 | hsa:3158 | 1 |
| hsa04211 | Longevity regulating pathway | 3\|118 | 103\|9228 | 0.1448961 | 0.6737286 | ENSG00000157613/ENSG00000109819/ENSG00000132170 | CREB3L1/PPARGC1A/PPARG | hsa:90993/hsa:10891/hsa:5468 | 3 |
| hsa00010 | Glycolysis / Gluconeogenesis | 3\|118 | 104\|9228 | 0.1478916 | 0.6737286 | ENSG00000259583/ENSG00000184254/ENSG00000186197 | AC015712.2/ALDH1A3/EDARADD | hsa:220/hsa:220/hsa:2023 | 3 |
| hsa05410 | Hypertrophic cardiomyopathy (HCM) | 3\|118 | 106\|9228 | 0.1539431 | 0.6802182 | ENSG00000078401/ENSG00000092969/ENSG00000115221 | EDN1/TGFB2/ITGB6 | hsa:1906/hsa:7042/hsa:3694 | 3 |
| hsa04923 | Regulation of lipolysis in adipocytes | 2\|118 | 55\|9228 | 0.1559525 | 0.6802182 | ENSG00000185532/ENSG00000135346 | PRKG1/CGA | hsa:5592/hsa:1081 | 2 |
| hsa04961 | Endocrine and other factor-regulated calcium reabsorption | 2\|118 | 56\|9228 | 0.1604468 | 0.6852416 | ENSG00000197959/ENSG00000168398 | DNM3/BDKRB2 | hsa:26052/hsa:624 | 2 |
| hsa04950 | Maturity onset diabetes of the young | 1\|118 | 14\|9228 | 0.1649826 | 0.6902331 | ENSG00000164749 | HNF4G | hsa:3174 | 1 |
| hsa04380 | Osteoclast differentiation | 3\|118 | 116\|9228 | 0.1852936 | 0.7431986 | ENSG00000092969/ENSG00000116701/ENSG00000132170 | TGFB2/NCF2/PPARG | hsa:7042/hsa:4688/hsa:5468 | 3 |
| hsa00480 | Glutathione metabolism | 2\|118 | 62\|9228 | 0.1878681 | 0.7431986 | ENSG00000213366/ENSG00000241720 | GSTM2/AL158847.1 | hsa:2946/hsa:2947 | 2 |
| hsa05134 | Legionellosis | 3\|118 | 117\|9228 | 0.1885187 | 0.7431986 | ENSG00000169429/ENSG00000081041/ENSG00000137462 | CXCL8/CXCL2/TLR2 | hsa:3576/hsa:2920/hsa:7097 | 3 |
| hsa05165 | Human papillomavirus infection | 7\|118 | 377\|9228 | 0.2074771 | 0.7902472 | ENSG00000157613/ENSG00000058085/ENSG00000188153/ENSG00000169031/ENSG00000135862/ENSG00000053747/ENSG00000115221 | CREB3L1/LAMC2/COL4A5/COL4A3/LAMC1/LAMA3/ITGB6 | hsa:90993/hsa:3918/hsa:1287/hsa:1285/hsa:3915/hsa:3909/hsa:3694 | 7 |
| hsa04922 | Glucagon signaling pathway | 3\|118 | 123\|9228 | 0.2081627 | 0.7902472 | ENSG00000157613/ENSG00000109819/ENSG00000275993 | CREB3L1/PPARGC1A/SIK1B | hsa:90993/hsa:10891/hsa:102724428 | 3 |
| hsa04929 | GnRH secretion | 2\|118 | 68\|9228 | 0.2158793 | 0.8046408 | ENSG00000135346/ENSG00000136928 | CGA/GABBR2 | hsa:1081/hsa:9568 | 2 |
| hsa00590 | Arachidonic acid metabolism | 2\|118 | 71\|9228 | 0.2300308 | 0.8109279 | ENSG00000256612/ENSG00000188257 | CYP2B7P/PLA2G2A | hsa:1555/hsa:5320 | 2 |
| hsa04390 | Hippo signaling pathway | 4\|118 | 193\|9228 | 0.2336133 | 0.8109279 | ENSG00000118523/ENSG00000092969/ENSG00000109321/ENSG00000023445 | CTGF/TGFB2/AREG/BIRC3 | hsa:1490/hsa:7042/hsa:374/hsa:330 | 4 |
| hsa04977 | Vitamin digestion and absorption | 1\|118 | 21\|9228 | 0.2370459 | 0.8109279 | ENSG00000086205 | FOLH1 | hsa:2346 | 1 |
| hsa04918 | Thyroid hormone synthesis | 2\|118 | 73\|9228 | 0.2395007 | 0.8109279 | ENSG00000157613/ENSG00000135346 | CREB3L1/CGA | hsa:90993/hsa:1081 | 2 |
| hsa04976 | Bile secretion | 2\|118 | 73\|9228 | 0.2395007 | 0.8109279 | ENSG00000125257/ENSG00000080493 | ABCC4/SLC4A4 | hsa:10257/hsa:8671 | 2 |
| hsa05164 | Influenza A | 4\|118 | 196\|9228 | 0.2418980 | 0.8109279 | ENSG00000137648/ENSG00000169429/ENSG00000111335/ENSG00000134321 | TMPRSS4/CXCL8/OAS2/RSAD2 | hsa:56649/hsa:3576/hsa:4939/hsa:91543 | 4 |
| hsa04152 | AMPK signaling pathway | 3\|118 | 134\|9228 | 0.2452562 | 0.8109279 | ENSG00000157613/ENSG00000109819/ENSG00000132170 | CREB3L1/PPARGC1A/PPARG | hsa:90993/hsa:10891/hsa:5468 | 3 |
| hsa04662 | B cell receptor signaling pathway | 2\|118 | 76\|9228 | 0.2537404 | 0.8165085 | ENSG00000152689/ENSG00000070190 | RASGRP3/DAPP1 | hsa:25780/hsa:27071 | 2 |
| hsa04727 | GABAergic synapse | 2\|118 | 78\|9228 | 0.2632467 | 0.8165085 | ENSG00000136928/ENSG00000139112 | GABBR2/GABARAPL1 | hsa:9568/hsa:23710 | 2 |
| hsa04360 | Axon guidance | 4\|118 | 204\|9228 | 0.2643080 | 0.8165085 | ENSG00000074527/ENSG00000112902/ENSG00000196220/ENSG00000172602 | NTN4/SEMA5A/SRGAP3/RND1 | hsa:59277/hsa:9037/hsa:9901/hsa:27289 | 4 |
| hsa04022 | cGMP-PKG signaling pathway | 4\|118 | 207\|9228 | 0.2728153 | 0.8165085 | ENSG00000185532/ENSG00000157613/ENSG00000116741/ENSG00000168398 | PRKG1/CREB3L1/RGS2/BDKRB2 | hsa:5592/hsa:90993/hsa:5997/hsa:624 | 4 |
| hsa03320 | PPAR signaling pathway | 2\|118 | 81\|9228 | 0.2775093 | 0.8165085 | ENSG00000134240/ENSG00000132170 | HMGCS2/PPARG | hsa:3158/hsa:5468 | 2 |
| hsa05162 | Measles | 3\|118 | 144\|9228 | 0.2798246 | 0.8165085 | ENSG00000114423/ENSG00000111335/ENSG00000137462 | CBLB/OAS2/TLR2 | hsa:868/hsa:4939/hsa:7097 | 3 |
| hsa00534 | Glycosaminoglycan biosynthesis - heparan sulfate / heparin | 1\|118 | 26\|9228 | 0.2847101 | 0.8165085 | ENSG00000002587 | HS3ST1 | hsa:9957 | 1 |
| hsa05320 | Autoimmune thyroid disease | 1\|118 | 26\|9228 | 0.2847101 | 0.8165085 | ENSG00000135346 | CGA | hsa:1081 | 1 |
| hsa00592 | alpha-Linolenic acid metabolism | 1\|118 | 27\|9228 | 0.2938825 | 0.8165085 | ENSG00000188257 | PLA2G2A | hsa:5320 | 1 |
| hsa04620 | Toll-like receptor signaling pathway | 2\|118 | 85\|9228 | 0.2965003 | 0.8165085 | ENSG00000169429/ENSG00000137462 | CXCL8/TLR2 | hsa:3576/hsa:7097 | 2 |
| hsa04974 | Protein digestion and absorption | 2\|118 | 85\|9228 | 0.2965003 | 0.8165085 | ENSG00000188153/ENSG00000169031 | COL4A5/COL4A3 | hsa:1287/hsa:1285 | 2 |
| hsa04964 | Proximal tubule bicarbonate reclamation | 1\|118 | 28\|9228 | 0.3029382 | 0.8165085 | ENSG00000080493 | SLC4A4 | hsa:8671 | 1 |
| hsa05167 | Kaposi sarcoma-associated herpesvirus infection | 4\|118 | 220\|9228 | 0.3101698 | 0.8165085 | ENSG00000169429/ENSG00000159200/ENSG00000139112/ENSG00000081041 | CXCL8/RCAN1/GABARAPL1/CXCL2 | hsa:3576/hsa:1827/hsa:23710/hsa:2920 | 4 |
| hsa04115 | p53 signaling pathway | 2\|118 | 89\|9228 | 0.3154207 | 0.8165085 | ENSG00000146674/ENSG00000206075 | IGFBP3/SERPINB5 | hsa:3486/hsa:5268 | 2 |
| hsa05211 | Renal cell carcinoma | 2\|118 | 90\|9228 | 0.3201350 | 0.8165085 | ENSG00000116016/ENSG00000092969 | EPAS1/TGFB2 | hsa:2034/hsa:7042 | 2 |
| hsa00983 | Drug metabolism - other enzymes | 2\|118 | 91\|9228 | 0.3248417 | 0.8165085 | ENSG00000213366/ENSG00000241720 | GSTM2/AL158847.1 | hsa:2946/hsa:2947 | 2 |
| hsa05120 | Epithelial cell signaling in Helicobacter pylori infection | 2\|118 | 91\|9228 | 0.3248417 | 0.8165085 | ENSG00000169429/ENSG00000081041 | CXCL8/CXCL2 | hsa:3576/hsa:2920 | 2 |
| hsa00500 | Starch and sucrose metabolism | 1\|118 | 31\|9228 | 0.3294203 | 0.8165085 | ENSG00000165434 | PGM2L1 | hsa:283209 | 1 |
| hsa00591 | Linoleic acid metabolism | 1\|118 | 31\|9228 | 0.3294203 | 0.8165085 | ENSG00000188257 | PLA2G2A | hsa:5320 | 1 |
| hsa00900 | Terpenoid backbone biosynthesis | 1\|118 | 31\|9228 | 0.3294203 | 0.8165085 | ENSG00000134240 | HMGCS2 | hsa:3158 | 1 |
| hsa04713 | Circadian entrainment | 2\|118 | 94\|9228 | 0.3389109 | 0.8165085 | ENSG00000185532/ENSG00000108551 | PRKG1/RASD1 | hsa:5592/hsa:51655 | 2 |
| hsa04972 | Pancreatic secretion | 2\|118 | 94\|9228 | 0.3389109 | 0.8165085 | ENSG00000080493/ENSG00000188257 | SLC4A4/PLA2G2A | hsa:8671/hsa:5320 | 2 |
| hsa04371 | Apelin signaling pathway | 3\|118 | 161\|9228 | 0.3394195 | 0.8165085 | ENSG00000118523/ENSG00000109819/ENSG00000139112 | CTGF/PPARGC1A/GABARAPL1 | hsa:1490/hsa:10891/hsa:23710 | 3 |
| hsa00650 | Butanoate metabolism | 1\|118 | 33\|9228 | 0.3465182 | 0.8165085 | ENSG00000134240 | HMGCS2 | hsa:3158 | 1 |
| hsa01523 | Antifolate resistance | 1\|118 | 33\|9228 | 0.3465182 | 0.8165085 | ENSG00000125257 | ABCC4 | hsa:10257 | 1 |
| hsa00512 | Mucin type O-glycan biosynthesis | 1\|118 | 35\|9228 | 0.3631839 | 0.8434970 | ENSG00000144278 | GALNT13 | hsa:114805 | 1 |
| hsa04916 | Melanogenesis | 2\|118 | 102\|9228 | 0.3759640 | 0.8434970 | ENSG00000078401/ENSG00000157613 | EDN1/CREB3L1 | hsa:1906/hsa:90993 | 2 |
| hsa04520 | Adherens junction | 2\|118 | 103\|9228 | 0.3805396 | 0.8434970 | ENSG00000173482/ENSG00000127329 | PTPRM/PTPRB | hsa:5797/hsa:5787 | 2 |
| hsa05020 | Prion diseases | 1\|118 | 38\|9228 | 0.3873953 | 0.8434970 | ENSG00000135862 | LAMC1 | hsa:3915 | 1 |
| hsa04062 | Chemokine signaling pathway | 3\|118 | 175\|9228 | 0.3883730 | 0.8434970 | ENSG00000169429/ENSG00000006606/ENSG00000081041 | CXCL8/CCL26/CXCL2 | hsa:3576/hsa:10344/hsa:2920 | 3 |
| hsa03018 | RNA degradation | 2\|118 | 105\|9228 | 0.3896488 | 0.8434970 | ENSG00000159388/ENSG00000186197 | BTG2/EDARADD | hsa:7832/hsa:2023 | 2 |
| hsa00564 | Glycerophospholipid metabolism | 2\|118 | 107\|9228 | 0.3986994 | 0.8434970 | ENSG00000188257/ENSG00000087085 | PLA2G2A/ACHE | hsa:5320/hsa:43 | 2 |
| hsa04975 | Fat digestion and absorption | 1\|118 | 40\|9228 | 0.4030269 | 0.8434970 | ENSG00000188257 | PLA2G2A | hsa:5320 | 1 |
| hsa04725 | Cholinergic synapse | 2\|118 | 108\|9228 | 0.4032018 | 0.8434970 | ENSG00000157613/ENSG00000087085 | CREB3L1/ACHE | hsa:90993/hsa:43 | 2 |
| hsa05210 | Colorectal cancer | 2\|118 | 108\|9228 | 0.4032018 | 0.8434970 | ENSG00000092969/ENSG00000109321 | TGFB2/AREG | hsa:7042/hsa:374 | 2 |
| hsa04010 | MAPK signaling pathway | 5\|118 | 331\|9228 | 0.4175570 | 0.8434970 | ENSG00000152689/ENSG00000128052/ENSG00000092969/ENSG00000109321/ENSG00000120875 | RASGRP3/KDR/TGFB2/AREG/DUSP4 | hsa:25780/hsa:3791/hsa:7042/hsa:374/hsa:1846 | 5 |
| hsa04136 | Autophagy - other | 1\|118 | 42\|9228 | 0.4182629 | 0.8434970 | ENSG00000139112 | GABARAPL1 | hsa:23710 | 1 |
| hsa04666 | Fc gamma R-mediated phagocytosis | 2\|118 | 112\|9228 | 0.4210508 | 0.8434970 | ENSG00000078053/ENSG00000277443 | AMPH/MARCKS | hsa:273/hsa:4082 | 2 |
| hsa05414 | Dilated cardiomyopathy (DCM) | 2\|118 | 112\|9228 | 0.4210508 | 0.8434970 | ENSG00000092969/ENSG00000115221 | TGFB2/ITGB6 | hsa:7042/hsa:3694 | 2 |
| hsa05206 | MicroRNAs in cancer | 3\|118 | 185\|9228 | 0.4228443 | 0.8434970 | ENSG00000092969/ENSG00000206075/ENSG00000277443 | TGFB2/SERPINB5/MARCKS | hsa:7042/hsa:5268/hsa:4082 | 3 |
| hsa04928 | Parathyroid hormone synthesis, secretion and action | 2\|118 | 113\|9228 | 0.4254714 | 0.8434970 | ENSG00000157613/ENSG00000137745 | CREB3L1/MMP13 | hsa:90993/hsa:4322 | 2 |
| hsa05216 | Thyroid cancer | 1\|118 | 44\|9228 | 0.4331133 | 0.8434970 | ENSG00000132170 | PPARG | hsa:5468 | 1 |
| hsa04931 | Insulin resistance | 2\|118 | 116\|9228 | 0.4386281 | 0.8434970 | ENSG00000157613/ENSG00000109819 | CREB3L1/PPARGC1A | hsa:90993/hsa:10891 | 2 |
| hsa04742 | Taste transduction | 1\|118 | 45\|9228 | 0.4403969 | 0.8434970 | ENSG00000136928 | GABBR2 | hsa:9568 | 1 |
| hsa04215 | Apoptosis - multiple species | 1\|118 | 46\|9228 | 0.4475877 | 0.8434970 | ENSG00000023445 | BIRC3 | hsa:330 | 1 |
| hsa04014 | Ras signaling pathway | 4\|118 | 269\|9228 | 0.4523057 | 0.8434970 | ENSG00000152689/ENSG00000128052/ENSG00000143344/ENSG00000188257 | RASGRP3/KDR/RGL1/PLA2G2A | hsa:25780/hsa:3791/hsa:23179/hsa:5320 | 4 |
| hsa04979 | Cholesterol metabolism | 1\|118 | 47\|9228 | 0.4546868 | 0.8434970 | ENSG00000101670 | LIPG | hsa:9388 | 1 |
| hsa05030 | Cocaine addiction | 1\|118 | 47\|9228 | 0.4546868 | 0.8434970 | ENSG00000157613 | CREB3L1 | hsa:90993 | 1 |
| hsa05219 | Bladder cancer | 1\|118 | 48\|9228 | 0.4616956 | 0.8434970 | ENSG00000169429 | CXCL8 | hsa:3576 | 1 |
| hsa04935 | Growth hormone synthesis, secretion and action | 2\|118 | 122\|9228 | 0.4644464 | 0.8434970 | ENSG00000157613/ENSG00000146674 | CREB3L1/IGFBP3 | hsa:90993/hsa:3486 | 2 |
| hsa04740 | Olfactory transduction | 2\|118 | 123\|9228 | 0.4686825 | 0.8434970 | ENSG00000185532/ENSG00000116741 | PRKG1/RGS2 | hsa:5592/hsa:5997 | 2 |
| hsa00514 | Other types of O-glycan biosynthesis | 1\|118 | 50\|9228 | 0.4754461 | 0.8434970 | ENSG00000144278 | GALNT13 | hsa:114805 | 1 |
| hsa00565 | Ether lipid metabolism | 1\|118 | 51\|9228 | 0.4821902 | 0.8434970 | ENSG00000188257 | PLA2G2A | hsa:5320 | 1 |
| hsa04913 | Ovarian steroidogenesis | 1\|118 | 51\|9228 | 0.4821902 | 0.8434970 | ENSG00000135346 | CGA | hsa:1081 | 1 |
| hsa00600 | Sphingolipid metabolism | 1\|118 | 52\|9228 | 0.4888483 | 0.8434970 | ENSG00000177076 | ACER2 | hsa:340485 | 1 |
| hsa00970 | Aminoacyl-tRNA biosynthesis | 1\|118 | 52\|9228 | 0.4888483 | 0.8434970 | novel.282 |  | hsa:54938 | 1 |
| hsa05225 | Hepatocellular carcinoma | 3\|118 | 205\|9228 | 0.4896397 | 0.8434970 | ENSG00000213366/ENSG00000092969/ENSG00000241720 | GSTM2/TGFB2/AL158847.1 | hsa:2946/hsa:7042/hsa:2947 | 3 |
| hsa00140 | Steroid hormone biosynthesis | 1\|118 | 54\|9228 | 0.5019110 | 0.8541402 | ENSG00000101846 | STS | hsa:412 | 1 |
| hsa04071 | Sphingolipid signaling pathway | 2\|118 | 132\|9228 | 0.5058999 | 0.8541402 | ENSG00000177076/ENSG00000168398 | ACER2/BDKRB2 | hsa:340485/hsa:624 | 2 |
| hsa00250 | Alanine, aspartate and glutamate metabolism | 1\|118 | 55\|9228 | 0.5083176 | 0.8541402 | ENSG00000086205 | FOLH1 | hsa:2346 | 1 |
| hsa04730 | Long-term depression | 1\|118 | 56\|9228 | 0.5146425 | 0.8573643 | ENSG00000185532 | PRKG1 | hsa:5592 | 1 |
| hsa00330 | Arginine and proline metabolism | 1\|118 | 58\|9228 | 0.5270513 | 0.8573643 | ENSG00000130066 | SAT1 | hsa:6303 | 1 |
| hsa00280 | Valine, leucine and isoleucine degradation | 1\|118 | 59\|9228 | 0.5331373 | 0.8573643 | ENSG00000134240 | HMGCS2 | hsa:3158 | 1 |
| hsa04622 | RIG-I-like receptor signaling pathway | 1\|118 | 61\|9228 | 0.5450771 | 0.8573643 | ENSG00000169429 | CXCL8 | hsa:3576 | 1 |
| hsa04217 | Necroptosis | 3\|118 | 223\|9228 | 0.5464543 | 0.8573643 | ENSG00000162909/ENSG00000138378/ENSG00000023445 | CAPN2/STAT4/BIRC3 | hsa:824/hsa:6775/hsa:330 | 3 |
| hsa04120 | Ubiquitin mediated proteolysis | 3\|118 | 224\|9228 | 0.5495042 | 0.8573643 | ENSG00000114423/ENSG00000101871/ENSG00000023445 | CBLB/MID1/BIRC3 | hsa:868/hsa:4281/hsa:330 | 3 |
| hsa04670 | Leukocyte transendothelial migration | 2\|118 | 147\|9228 | 0.5640936 | 0.8573643 | ENSG00000179776/ENSG00000116701 | CDH5/NCF2 | hsa:1003/hsa:4688 | 2 |
| hsa05169 | Epstein-Barr virus infection | 3\|118 | 230\|9228 | 0.5675523 | 0.8573643 | ENSG00000168032/ENSG00000111335/ENSG00000137462 | ENTPD3/OAS2/TLR2 | hsa:956/hsa:4939/hsa:7097 | 3 |
| hsa04919 | Thyroid hormone signaling pathway | 2\|118 | 148\|9228 | 0.5677964 | 0.8573643 | ENSG00000159200/ENSG00000211448 | RCAN1/DIO2 | hsa:1827/hsa:1734 | 2 |
| hsa04927 | Cortisol synthesis and secretion | 1\|118 | 65\|9228 | 0.5680560 | 0.8573643 | ENSG00000157613 | CREB3L1 | hsa:90993 | 1 |
| hsa05031 | Amphetamine addiction | 1\|118 | 65\|9228 | 0.5680560 | 0.8573643 | ENSG00000157613 | CREB3L1 | hsa:90993 | 1 |
| hsa00561 | Glycerolipid metabolism | 1\|118 | 67\|9228 | 0.5791100 | 0.8573643 | ENSG00000101670 | LIPG | hsa:9388 | 1 |
| hsa04066 | HIF-1 signaling pathway | 2\|118 | 152\|9228 | 0.5823826 | 0.8573643 | ENSG00000078401/ENSG00000186197 | EDN1/EDARADD | hsa:1906/hsa:2023 | 2 |
| hsa00240 | Pyrimidine metabolism | 1\|118 | 68\|9228 | 0.5845313 | 0.8573643 | ENSG00000168032 | ENTPD3 | hsa:956 | 1 |
| hsa04370 | VEGF signaling pathway | 1\|118 | 69\|9228 | 0.5898834 | 0.8573643 | ENSG00000128052 | KDR | hsa:3791 | 1 |
| hsa04924 | Renin secretion | 1\|118 | 69\|9228 | 0.5898834 | 0.8573643 | ENSG00000078401 | EDN1 | hsa:1906 | 1 |
| hsa05133 | Pertussis | 1\|118 | 69\|9228 | 0.5898834 | 0.8573643 | ENSG00000169429 | CXCL8 | hsa:3576 | 1 |
| hsa05205 | Proteoglycans in cancer | 3\|118 | 238\|9228 | 0.5909274 | 0.8573643 | ENSG00000128052/ENSG00000092969/ENSG00000137462 | KDR/TGFB2/TLR2 | hsa:3791/hsa:7042/hsa:7097 | 3 |
| hsa05032 | Morphine addiction | 1\|118 | 70\|9228 | 0.5951672 | 0.8573643 | ENSG00000136928 | GABBR2 | hsa:9568 | 1 |
| hsa04015 | Rap1 signaling pathway | 3\|118 | 240\|9228 | 0.5966448 | 0.8573643 | ENSG00000152689/ENSG00000128052/ENSG00000082074 | RASGRP3/KDR/FYB1 | hsa:25780/hsa:3791/hsa:2533 | 3 |
| hsa04920 | Adipocytokine signaling pathway | 1\|118 | 71\|9228 | 0.6003834 | 0.8573643 | ENSG00000109819 | PPARGC1A | hsa:10891 | 1 |
| hsa05135 | Yersinia infection | 2\|118 | 164\|9228 | 0.6239744 | 0.8573643 | ENSG00000169429/ENSG00000082074 | CXCL8/FYB1 | hsa:3576/hsa:2533 | 2 |
| hsa04068 | FoxO signaling pathway | 2\|118 | 165\|9228 | 0.6272937 | 0.8573643 | ENSG00000092969/ENSG00000139112 | TGFB2/GABARAPL1 | hsa:7042/hsa:23710 | 2 |
| hsa04072 | Phospholipase D signaling pathway | 2\|118 | 165\|9228 | 0.6272937 | 0.8573643 | ENSG00000197959/ENSG00000169429 | DNM3/CXCL8 | hsa:26052/hsa:3576 | 2 |
| hsa04658 | Th1 and Th2 cell differentiation | 1\|118 | 77\|9228 | 0.6303104 | 0.8573643 | ENSG00000138378 | STAT4 | hsa:6775 | 1 |
| hsa04917 | Prolactin signaling pathway | 1\|118 | 77\|9228 | 0.6303104 | 0.8573643 | ENSG00000135346 | CGA | hsa:1081 | 1 |
| hsa04934 | Cushing syndrome | 2\|118 | 166\|9228 | 0.6305904 | 0.8573643 | ENSG00000157613/ENSG00000108551 | CREB3L1/RASD1 | hsa:90993/hsa:51655 | 2 |
| hsa04911 | Insulin secretion | 1\|118 | 78\|9228 | 0.6350774 | 0.8573643 | ENSG00000157613 | CREB3L1 | hsa:90993 | 1 |
| hsa04970 | Salivary secretion | 1\|118 | 78\|9228 | 0.6350774 | 0.8573643 | ENSG00000185532 | PRKG1 | hsa:5592 | 1 |
| hsa04144 | Endocytosis | 4\|118 | 343\|9228 | 0.6442534 | 0.8573643 | ENSG00000078053/ENSG00000197959/ENSG00000153071/ENSG00000114423 | AMPH/DNM3/DAB2/CBLB | hsa:273/hsa:26052/hsa:1601/hsa:868 | 4 |
| hsa04910 | Insulin signaling pathway | 2\|118 | 172\|9228 | 0.6499000 | 0.8573643 | ENSG00000109819/ENSG00000114423 | PPARGC1A/CBLB | hsa:10891/hsa:868 | 2 |
| hsa05160 | Hepatitis C | 2\|118 | 173\|9228 | 0.6530401 | 0.8573643 | ENSG00000111335/ENSG00000134321 | OAS2/RSAD2 | hsa:4939/hsa:91543 | 2 |
| hsa00310 | Lysine degradation | 1\|118 | 85\|9228 | 0.6667827 | 0.8573643 | ENSG00000008311 | AASS | hsa:10157 | 1 |
| hsa05152 | Tuberculosis | 2\|118 | 178\|9228 | 0.6684087 | 0.8573643 | ENSG00000092969/ENSG00000137462 | TGFB2/TLR2 | hsa:7042/hsa:7097 | 2 |
| hsa04137 | Mitophagy - animal | 2\|118 | 180\|9228 | 0.6744023 | 0.8573643 | ENSG00000139112/ENSG00000123240 | GABARAPL1/OPTN | hsa:23710/hsa:10133 | 2 |
| hsa04915 | Estrogen signaling pathway | 2\|118 | 180\|9228 | 0.6744023 | 0.8573643 | ENSG00000157613/ENSG00000136928 | CREB3L1/GABBR2 | hsa:90993/hsa:9568 | 2 |
| hsa04921 | Oxytocin signaling pathway | 2\|118 | 180\|9228 | 0.6744023 | 0.8573643 | ENSG00000159200/ENSG00000116741 | RCAN1/RGS2 | hsa:1827/hsa:5997 | 2 |
| hsa04721 | Synaptic vesicle cycle | 1\|118 | 87\|9228 | 0.6753287 | 0.8573643 | ENSG00000197959 | DNM3 | hsa:26052 | 1 |
| hsa04140 | Autophagy - animal | 3\|118 | 270\|9228 | 0.6761033 | 0.8573643 | ENSG00000139112/ENSG00000078804/ENSG00000062716 | GABARAPL1/TP53INP2/VMP1 | hsa:23710/hsa:58476/hsa:81671 | 3 |
| hsa04971 | Gastric acid secretion | 1\|118 | 88\|9228 | 0.6795198 | 0.8573643 | ENSG00000147606 | SLC26A7 | hsa:115111 | 1 |
| hsa04714 | Thermogenesis | 4\|118 | 360\|9228 | 0.6817092 | 0.8573643 | ENSG00000185532/ENSG00000157613/ENSG00000109819/ENSG00000132170 | PRKG1/CREB3L1/PPARGC1A/PPARG | hsa:5592/hsa:90993/hsa:10891/hsa:5468 | 4 |
| hsa04210 | Apoptosis | 2\|118 | 185\|9228 | 0.6890055 | 0.8612569 | ENSG00000162909/ENSG00000023445 | CAPN2/BIRC3 | hsa:824/hsa:330 | 2 |
| hsa04145 | Phagosome | 2\|118 | 192\|9228 | 0.7085504 | 0.8665350 | ENSG00000116701/ENSG00000137462 | NCF2/TLR2 | hsa:4688/hsa:7097 | 2 |
| hsa04750 | Inflammatory mediator regulation of TRP channels | 1\|118 | 96\|9228 | 0.7111737 | 0.8665350 | ENSG00000168398 | BDKRB2 | hsa:624 | 1 |
| hsa05212 | Pancreatic cancer | 1\|118 | 96\|9228 | 0.7111737 | 0.8665350 | ENSG00000092969 | TGFB2 | hsa:7042 | 1 |
| hsa01521 | EGFR tyrosine kinase inhibitor resistance | 1\|118 | 97\|9228 | 0.7149058 | 0.8665350 | ENSG00000128052 | KDR | hsa:3791 | 1 |
| hsa04660 | T cell receptor signaling pathway | 1\|118 | 98\|9228 | 0.7185901 | 0.8665350 | ENSG00000114423 | CBLB | hsa:868 | 1 |
| hsa05235 | PD-L1 expression and PD-1 checkpoint pathway in cancer | 1\|118 | 99\|9228 | 0.7222271 | 0.8665350 | ENSG00000137462 | TLR2 | hsa:7097 | 1 |
| hsa05220 | Chronic myeloid leukemia | 1\|118 | 100\|9228 | 0.7258176 | 0.8665350 | ENSG00000092969 | TGFB2 | hsa:7042 | 1 |
| hsa04350 | TGF-beta signaling pathway | 1\|118 | 103\|9228 | 0.7363151 | 0.8665350 | ENSG00000092969 | TGFB2 | hsa:7042 | 1 |
| hsa04925 | Aldosterone synthesis and secretion | 1\|118 | 103\|9228 | 0.7363151 | 0.8665350 | ENSG00000157613 | CREB3L1 | hsa:90993 | 1 |
| hsa04540 | Gap junction | 1\|118 | 104\|9228 | 0.7397250 | 0.8665350 | ENSG00000185532 | PRKG1 | hsa:5592 | 1 |
| hsa04912 | GnRH signaling pathway | 1\|118 | 104\|9228 | 0.7397250 | 0.8665350 | ENSG00000135346 | CGA | hsa:1081 | 1 |
| hsa04724 | Glutamatergic synapse | 1\|118 | 106\|9228 | 0.7464140 | 0.8682901 | ENSG00000164418 | GRIK2 | hsa:2898 | 1 |
| hsa05412 | Arrhythmogenic right ventricular cardiomyopathy (ARVC) | 1\|118 | 107\|9228 | 0.7496944 | 0.8682901 | ENSG00000115221 | ITGB6 | hsa:3694 | 1 |
| hsa04625 | C-type lectin receptor signaling pathway | 1\|118 | 111\|9228 | 0.7624002 | 0.8766618 | ENSG00000114423 | CBLB | hsa:868 | 1 |
| hsa05100 | Bacterial invasion of epithelial cells | 1\|118 | 112\|9228 | 0.7654754 | 0.8766618 | ENSG00000197959 | DNM3 | hsa:26052 | 1 |
| hsa05215 | Prostate cancer | 1\|118 | 115\|9228 | 0.7744663 | 0.8795795 | ENSG00000157613 | CREB3L1 | hsa:90993 | 1 |
| hsa05132 | Salmonella infection | 3\|118 | 317\|9228 | 0.7766044 | 0.8795795 | ENSG00000169429/ENSG00000023445/ENSG00000137462 | CXCL8/BIRC3/TLR2 | hsa:3576/hsa:330/hsa:7097 | 3 |
| hsa01230 | Biosynthesis of amino acids | 1\|118 | 118\|9228 | 0.7831152 | 0.8820804 | ENSG00000186197 | EDARADD | hsa:2023 | 1 |
| hsa04728 | Dopaminergic synapse | 1\|118 | 133\|9228 | 0.8216668 | 0.9152657 | ENSG00000157613 | CREB3L1 | hsa:90993 | 1 |
| hsa05163 | Human cytomegalovirus infection | 2\|118 | 251\|9228 | 0.8357877 | 0.9152657 | ENSG00000157613/ENSG00000169429 | CREB3L1/CXCL8 | hsa:90993/hsa:3576 | 2 |
| hsa04611 | Platelet activation | 1\|118 | 141\|9228 | 0.8393641 | 0.9152657 | ENSG00000185532 | PRKG1 | hsa:5592 | 1 |
| hsa04810 | Regulation of actin cytoskeleton | 2\|118 | 257\|9228 | 0.8454521 | 0.9152657 | ENSG00000168398/ENSG00000115221 | BDKRB2/ITGB6 | hsa:624/hsa:3694 | 2 |
| hsa04550 | Signaling pathways regulating pluripotency of stem cells | 1\|118 | 144\|9228 | 0.8455417 | 0.9152657 | ENSG00000105991 | HOXA1 | hsa:3198 | 1 |
| hsa04722 | Neurotrophin signaling pathway | 1\|118 | 145\|9228 | 0.8475481 | 0.9152657 | ENSG00000111348 | ARHGDIB | hsa:397 | 1 |
| hsa05166 | Human T-cell leukemia virus 1 infection | 2\|118 | 261\|9228 | 0.8516079 | 0.9152657 | ENSG00000157613/ENSG00000092969 | CREB3L1/TGFB2 | hsa:90993/hsa:7042 | 2 |
| hsa04630 | JAK-STAT signaling pathway | 1\|118 | 148\|9228 | 0.8534135 | 0.9152657 | ENSG00000138378 | STAT4 | hsa:6775 | 1 |
| hsa05226 | Gastric cancer | 1\|118 | 148\|9228 | 0.8534135 | 0.9152657 | ENSG00000092969 | TGFB2 | hsa:7042 | 1 |
| hsa00230 | Purine metabolism | 1\|118 | 153\|9228 | 0.8626960 | 0.9152657 | ENSG00000168032 | ENTPD3 | hsa:956 | 1 |
| hsa05131 | Shigellosis | 3\|118 | 375\|9228 | 0.8641410 | 0.9152657 | ENSG00000169429/ENSG00000162909/ENSG00000139112 | CXCL8/CAPN2/GABARAPL1 | hsa:3576/hsa:824/hsa:23710 | 3 |
| hsa04261 | Adrenergic signaling in cardiomyocytes | 1\|118 | 155\|9228 | 0.8662437 | 0.9152657 | ENSG00000157613 | CREB3L1 | hsa:90993 | 1 |
| hsa05130 | Pathogenic Escherichia coli infection | 2\|118 | 276\|9228 | 0.8727683 | 0.9152657 | ENSG00000169429/ENSG00000006453 | CXCL8/BAIAP2L1 | hsa:3576/hsa:55971 | 2 |
| hsa04142 | Lysosome | 1\|118 | 162\|9228 | 0.8779595 | 0.9152657 | ENSG00000119899 | SLC17A5 | hsa:26503 | 1 |
| hsa04110 | Cell cycle | 1\|118 | 163\|9228 | 0.8795480 | 0.9152657 | ENSG00000092969 | TGFB2 | hsa:7042 | 1 |
| hsa04310 | Wnt signaling pathway | 1\|118 | 169\|9228 | 0.8886571 | 0.9200743 | ENSG00000133067 | LGR6 | hsa:59352 | 1 |
| hsa01200 | Carbon metabolism | 1\|118 | 183\|9228 | 0.9073412 | 0.9346983 | ENSG00000186197 | EDARADD | hsa:2023 | 1 |
| hsa04020 | Calcium signaling pathway | 1\|118 | 202\|9228 | 0.9278195 | 0.9489214 | ENSG00000168398 | BDKRB2 | hsa:624 | 1 |
| hsa05034 | Alcoholism | 1\|118 | 205\|9228 | 0.9306138 | 0.9489214 | ENSG00000157613 | CREB3L1 | hsa:90993 | 1 |
| hsa04141 | Protein processing in endoplasmic reticulum | 1\|118 | 210\|9228 | 0.9350347 | 0.9489214 | ENSG00000162909 | CAPN2 | hsa:824 | 1 |
| hsa04932 | Non-alcoholic fatty liver disease (NAFLD) | 1\|118 | 225\|9228 | 0.9466897 | 0.9560167 | ENSG00000169429 | CXCL8 | hsa:3576 | 1 |
| hsa05170 | Human immunodeficiency virus 1 infection | 1\|118 | 242\|9228 | 0.9574091 | 0.9621023 | ENSG00000137462 | TLR2 | hsa:7097 | 1 |
| hsa05203 | Viral carcinogenesis | 1\|118 | 275\|9228 | 0.9724874 | 0.9724874 | ENSG00000157613 | CREB3L1 | hsa:90993 | 1 |
